# Outcomes for a custom-made anchor-like plate combined with cerclage in the treatment of inferior pole patellar fracture

**DOI:** 10.1186/s12891-022-05413-7

**Published:** 2022-05-14

**Authors:** Ming Li, Hongfei Qi, Teng Ma, Zhong Li, Cheng Ren, Qiang Huang, Hanzhong Xue, Yao Lu, Yanling Yang, Kun Zhang

**Affiliations:** 1 Department of Orthopaedics and Trauma, Hong Hui Hospital, Xi’an Jiaotong, University College of Medicine, No. 555, East Youyi Road, Xi’an, 710000 Shaanxi China; 2 Medical College of Yan’an University, No. 30, Guanghua Road, Baota District, Yan’an, 716000 Shaanxi China

**Keywords:** Patellar fractures, Bone Plates, Comminution, Marginal fracture

## Abstract

**Objective:**

An inferior pole fracture of the patella requires surgical treatment to restore the knee extension mechanism of the knee joint. Different from other types of patellar fractures, inferior pole fractures are usually comminuted, and other traditional fixation methods, such as tension band wiring, may not meet the fixation needs. We propose fixing inferior pole fractures of the patella with a custom-made anchor-like plate combined with cerclage and report the surgical outcomes.

**Material and methods:**

This is a retrospective clinical study. From June 2018 to August 2020, 21 patients with inferior patella fracture treated at Hong Hui Hospital Affiliated to Xi’an Jiaotong University received a custom-made anchor-like plate combined with cerclage. Complications of the surgical fixation methods and final knee function were used as the main outcome measures.

**Results:**

All fractures achieved good union, and the union time ranged from 8 to 12 weeks. No patients had serious complications, such as internal fixation failure or infection. The average duration of surgery of patients was 75.05 7.26 min, and the intraoperative blood loss was 60.099.49 ml. At the last follow-up, the range of motion of the knee was 120°-140°, with an average of 131.436.92°, the Bostman score was 27–30, and the Lysholm score ranged from 82 to 95. All patients showed good knee function one year after the operation.

**Conclusion:**

We used a modified T-shaped plate combined with cerclage technology to fix inferior fractures pole of the patella, providing reliable fixation, allowing early functional exercise of the knee joint, and providing patients with good knee joint function after surgery.

## Background

An inferior pole fracture of the patella will lead to destruction of the knee extension mechanism, which requires surgical treatment [1]. Inferior pole fracture of the patella accounts for approximately 9.3–22.4% of all patients with patella fracture [2, 3]. Such fractures are usually comminuted, and the fracture fragment is small. There are some difficulties in fixation and reduction. A variety of techniques have been used for this type of fracture, but no ideal method has been widely recognized. Tension band wiring (TBW) is one of the most classic fixation methods in patellar fractures. However, some studies have shown that this technology has the risk of internal fixation failure in elderly patients with comminuted fractures [4, 5], which means that the risk may be higher in inferior pole fractures. Cerclage fixation and suturing are used to fix fractures of the inferior pole of the patella; however, cerclage cannot effectively close the articular surface of the proximal patella, and there is a risk of debris entering the articular cavity [6]. A partial patellectomy with repair of the patellar tendon and anchor suturing has been used in the treatment of distal patellar fracture [7]. However, there may be a poor long-term prognosis after patellar resection, and approximately half of the patients will experience a decrease in quadriceps femoris strength [8].

There are many applications for plate fixation of patellar fractures, among which the most classic method is the basket plate [9, 10], which solves the problem of reduction and fixation of inferior pole fractures of the patella. In addition, a prospective cohort study showed that plate fixation had better functional results than TBW fixation in the treatment of patellar fractures [11]. Singer et al. [12] reported that the application of a low-profile mesh plate also has a good effect on comminuted patella fractures. Similar to other types of patellar fractures, the purpose of treatment is to restore the function of the quadriceps femoris, reconstruct the knee extension mechanism and promote early functional exercise [13]. Our centre uses a custom-made anchor-like plate combined with cerclage to fix fractures of the inferior pole of the patella and has the advantages of reliable fixation and simple operation. This study retrospectively analysed the clinical data of patients with inferior patellar fracture fixed with a custom-made anchor-like plate combined with cerclage at the Hong Hui Hospital Affiliated to Xi’an Jiaotong University from June 2018 to August 2020 and observed their clinical results.

## Patients and methods

### Inclusion and exclusion criteria

The inclusion criteria were as follows: 1. fresh fracture of the inferior patella; 2. diagnosed as AO/OTA 34-A1 by the first author; 3. the knee joint function was good before injury; and 4. the follow-up data were complete.

The exclusion criteria were as follows: 1. patients with fracture of the distal femur or proximal tibia; 2. time from injury to surgery> 2 weeks; 3. patients with previous history of knee trauma; 4. patients treated with other fixation methods; and 5. patients who were lost to follow-up or were followed up for less than 12 months.

### General information

**Fig. 1:**
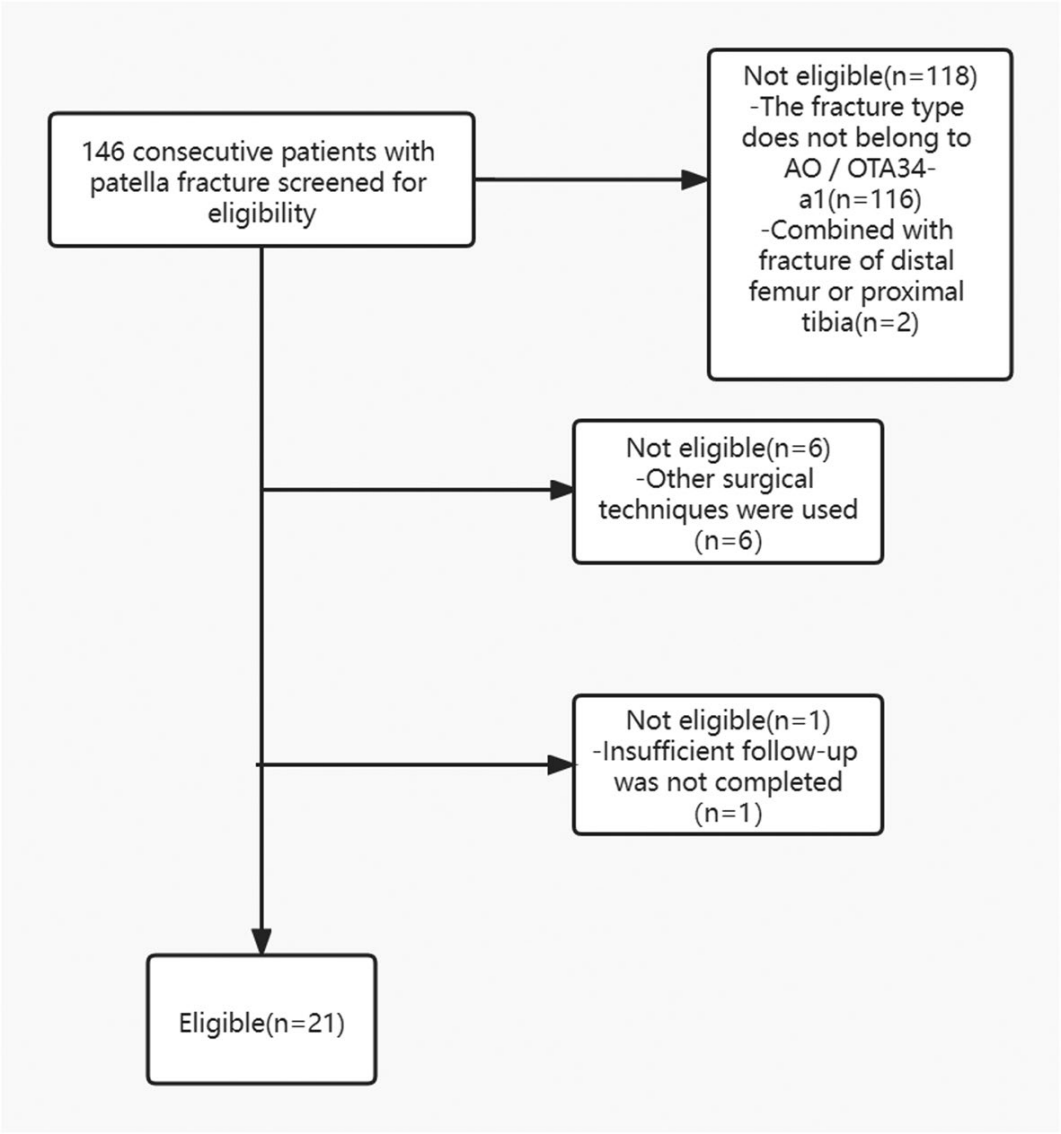
Flow diagram of patient recruitment and retention

From June 2018 to August 2020, 146 patients with patellar fractures were treated at Hong Hui Hospital of Xi’an Jiaotong University, including 30 cases (AO/OTA34-a1) that met the inclusion criteria; of these, 6 cases were treated with other operations, 2 cases were complicated with fractures of the distal femur or proximal tibia; and one case did not complete enough follow-up. According to the inclusion and exclusion criteria, 21 patients with inferior patella fracture treated with a custom-made anchor-like plate and cerclage were finally included in our clinical study. The patient inclusion flow chart is shown in Fig. 1, and the demographic characteristics and general conditions of the patients are shown in Table 1. There were 21 patients, including 14 males and 7 females. The age range was 32–61 years old, with an average age of 43.907.95 years. Eleven patients had fall injuries, and 10 patients had traffic accident injuries. The patients included in the study underwent X-ray film, computed tomography (CT), and three-dimensional reconstruction CT after admission. The diameter of the inferior patellar fracture fragment was measured by three-dimensional CT. The results showed that the diameter of the inferior patellar fracture fragments of the 21 patients ranged from 9.0 to 13.2 mm, with an average of 10.821.39 mm. This study was approved by the ethics committee of the Hong Hui Hospital Affiliated to Xi’an Jiaotong University. All patients included in the study signed informed consent forms, and the surgery was performed by Dr. Li Ming for all patients.

**Table 1:** Patient demographics and basic information

Patient	Age (years)	Sex	Cause of trauma	Average length of inferior pole fracture fragments (mm)	Hospitalization (days)	Follow-up periods (months)
1	38	Male	Traffic Accident	8.0	5	12
2	42	Female	Traffic Accident	8.9	5	12
3	32	Male	Fall	12.4	6	14
4	46	Female	Traffic Accident	9.1	5	12
5	34	Male	Fall	11.3	5	12
6	57	Male	Fall	9.8	8	12
7	53	Female	Traffic Accident	12.1	5	13
8	45	Male	Fall	11.7	5	12
9	61	Male	Traffic Accident	10.3	7	14
10	38	Male	Fall	9.7	4	12
11	40	Male	Traffic Accident	11.3	5	14
12	36	Female	Traffic Accident	12.4	4	12
13	47	Male	Traffic Accident	9.5	5	12
14	52	Male	Fall	11.3	7	14
15	49	Female	Fall	13.2	4	12
16	36	Male	Traffic Accident	11.8	5	14
17	42	Male	Fall	9.6	4	12
18	44	Female	Traffic Accident	10.3	6	12
19	53	Male	Fall	10.7	7	14
20	36	Female	Fall	11.2	5	12
21	41	Male	Fall	12.6	4	12

### Surgical methods

**Fig. 2:**
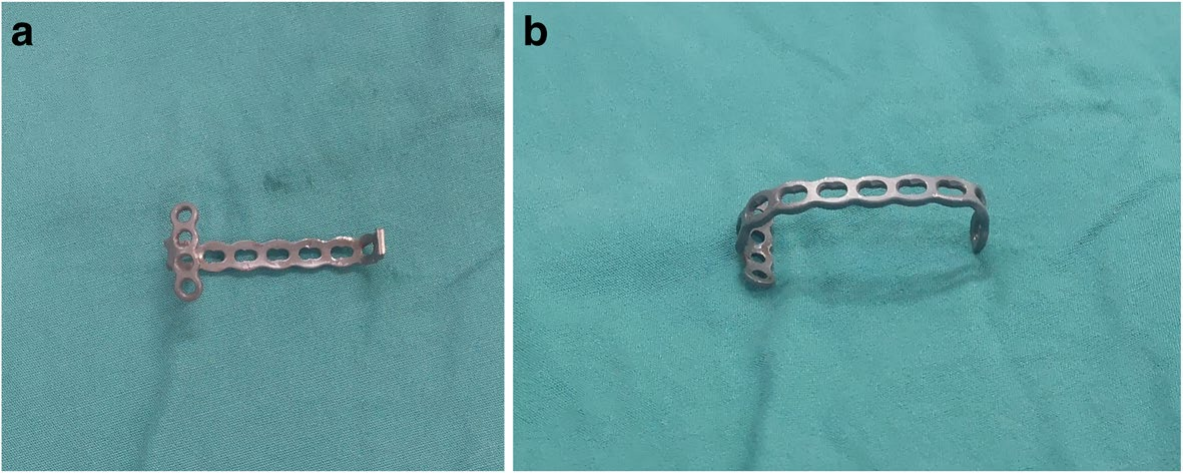
Custom-made anchor-like plate made by modifying a T-shaped plate

In our study, the anchor-like plate used for fixation was made by modifying a T-shaped plate (Fig. 2; T-shaped steel plate produced by Tianjin Zhengtian Company).

**Fig. 3:**
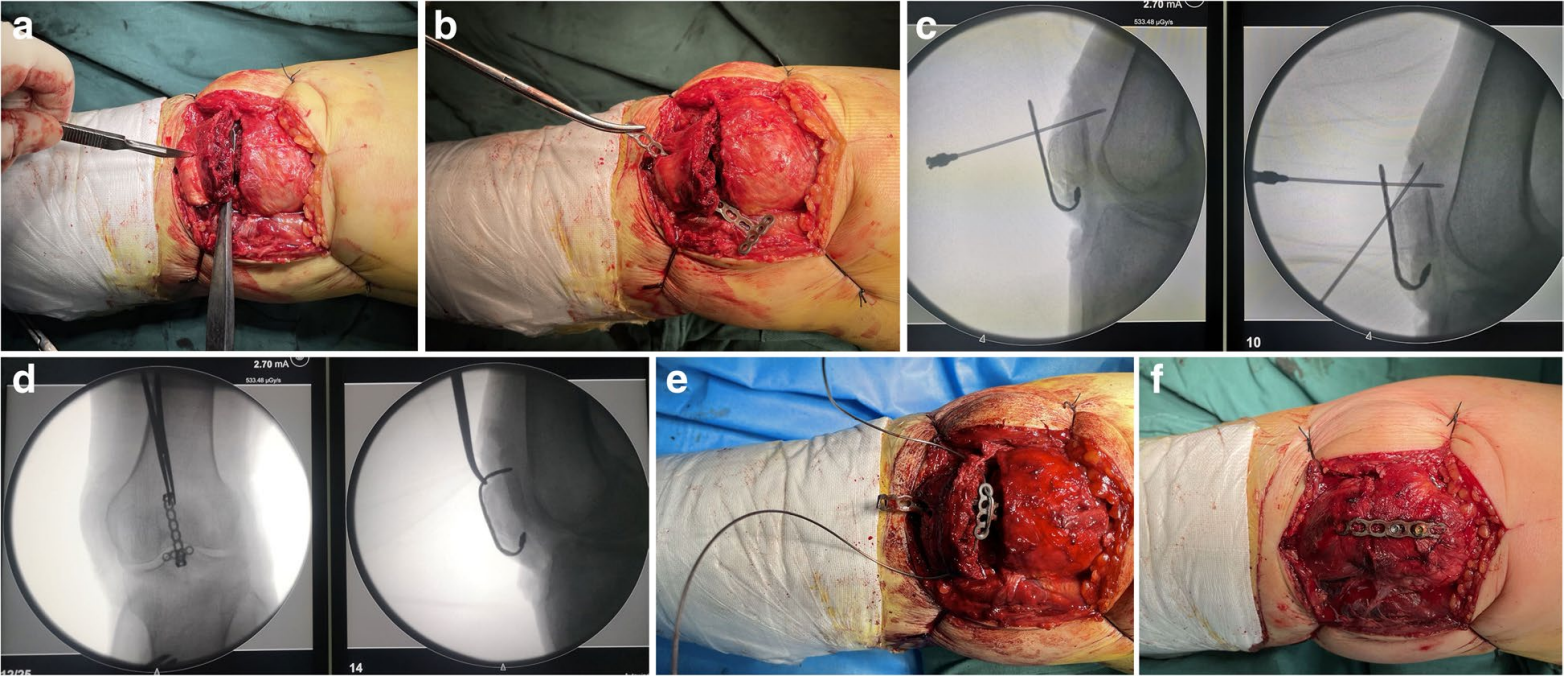
Intraoperative situation with the custom-made anchor-like plate in the treatment of lower patellar pole fracture. **a** A scalpel was used to cut a small opening in the patellar tendon at the lower pole. **b** The plate was passed through the small opening in the patellar tendon. **c-d** The plate was further shaped during the operation. **e** Additional cerclage of the combined steel wire. **f** After the hooping is pressurized, it is fixed with screws in turn

After satisfactory anaesthesia was achieved, the patient was placed in the supine position, antibiotics were administered prophylactically, and a tourniquet was placed at the proximal thigh. A flap was opened through a longitudinal incision on the midline of the knee to expose the fracture and remove the blood clot at the broken end of the fracture. In this process, stripping of the anterior patellar fascia and periosteum was avoided to prevent separation of the fracture fragment. The fracture type and displacement degree were determined. Then, a small opening was made on the surface of the patellar tendon connecting the lower pole of the patella with a sharp knife, and the shaped T-shaped steel plate was passed through the patellar tendon through this small opening to firmly fix the T-shaped end of the steel plate to the surface of the lower pole fracture block (Fig. 3a-b). At this time, the patella tendon moved in the direction in which the plate was pulled. During this operation, we not only fix the lower pole fracture but also offset the pulling effect of the patella tendon on the inferior pole fracture fragment. The width of the T-shaped end of the plate must exceed the width of the distal fracture fragments to prevent the fracture fragments from leaking from the edge of the plate. The angle of the plate needs to be further shaped during the operation to make the plate fit the surface of the patella, and the position of the plate needs to ensure that the T-shaped end of the plate is close to the surface of the fracture block of the lower pole (Fig. 3c-d); the other end is close to the surface of the upper pole of the patella (the plate is cut to the appropriate length). Then, a titanium cable or steel wire with a diameter of 1.0 mm was passed through the T-end of the plate and fixed by cerclage fixation alternately through the soft tissue around the patella with the help of a lumbar puncture needle (Fig. 3e). After cerclage fixation, the titanium cable or steel wire was tightened and pressurized, and the plate was fixed on the patella surface with screws in turn. All screws are 2.7 system screws. Generally, we suggest that the screw perpendicular to the upper surface of the patella is cortical screw, and the rest are locking head; All screws were fixed with uni-cortical (Fig. 3f). C-arm fluoroscopy was used to assess the fracture reduction; the knee joint was bent, and the stability of the fracture fragment fixation was observed. After confirming that the fracture fixation was satisfactory, the drainage tube was retained in the wound, and the wound was closed layer by layer.

### Outcome measures

The main outcomes of this study were complications of the surgical fixation methods (internal fixation failure/displacement, breakage of internal fixation, and infection) and knee function. The secondary outcomes were the duration of surgery, intraoperative blood loss and fracture union time. Intraoperative blood loss was estimated by the weight of the surgical sponge and measuring the amount of blood collected by the suction tank. On the second day after the operation, the dressing was opened to observe the wound, the drainage tube was removed, and anteroposterior (A-P) and lateral X-ray and CT examinations of the knee joint were completed. The wound was cleaned, and the dressing was changed every 3 days to observe wound healing. Patients do not need to wear braces after operation. They can carry out passive activities of the knee joint after operation, and can start active activities of the knee joint 2 weeks after operation. After discharge, complete X-ray reexamination was performed every 4 weeks to observe the functional recovery of the knee joint and guide patients in carrying out functional exercise of the knee joint. The standard of fracture healing was based on the results of the radiographic examination and clinical outcomes. The outcomes of the radiographic examination show that the fragment end of the fracture healed and that there was no fracture line, which is considered to indicate fracture healing. The clinical results showed that the patient could walk independently and normally without knee pain. The fracture was considered to be healed. At the last follow-up, the range of motion of the patient’s knee was measured with a standard protractor. Knee function was evaluated by the Bostman score and the Lysholm score.

## Results

All patients included in the study completed at least 12 months of follow-up. Finally, all patients’ wounds and fractures achieved good union, and no patients reported serious complications, such as implant failure or any infection (Table 2). The average duration of surgery was 75.057.26 (64–87) minutes; The average intraoperative blood loss was 60.099.49 (49–82) ml. The time of fracture union was 9.331.59 (8–12) weeks. At the last follow-up, the range of motion of the knee was 120°-140°, with an average of 131.436.92°; 16 patients recovered the range of motion relative to the contralateral knee. Regarding the functional outcomes of the knee joint, all patients showed excellent or good results one year after the operation. The Bostman score ranged from 27 to 30, with an average of 28.671.24; the Lysholm score ranged from 82 to 95, with an average of 89.194.45.

**Table 2:** Patient clinical outcomes

Patient	Duration of Surgery (min)	Intraoperative Blood Loss (ml)	Union Time (week)	Final ROM	Bostman Score	Lysholm Score
1	72	56	8	0–140°	28	92
2	82	67	10	0–130°	28	84
3	68	62	8	0–140°	30	95
4	79	74	8	0–140°	30	90
5	85	82	8	0–135°	30	95
6	71	50	12	0–120°	27	90
7	68	49	10	0–125°	28	82
8	87	51	8	0–135°	30	86
9	83	62	12	0–120°	27	82
10	74	58	8	0–135°	30	90
11	66	60	8	0–130°	28	88
12	72	76	10	0–135°	28	92
13	82	49	10	0–130°	27	95
14	69	53	12	0–120°	30	86
15	77	63	8	0–120°	28	84
16	80	58	8	0–135°	30	90
17	72	61	10	0–130°	28	84
18	64	55	8	0–135°	27	95
19	65	72	12	0–140°	30	95
20	74	54	8	0–130°	28	90
21	86	50	10	0–135°	30	88

**Fig. 4:**
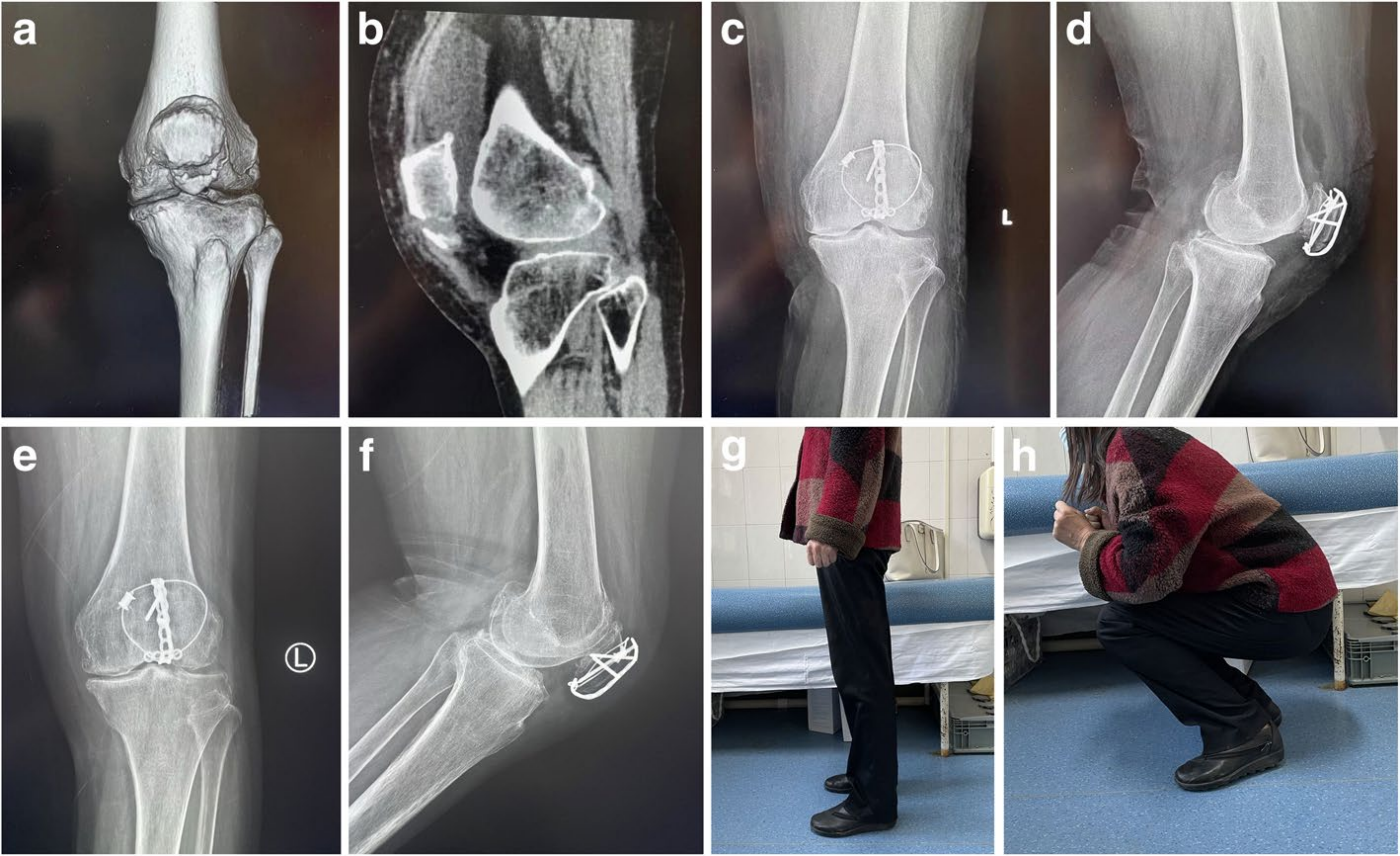
A 53-year-old male suffered a fracture of the inferior pole of the left patella due to a fall. **a**-**b** Preoperative three-dimensional CT; **c**-**d** anteroposterior and lateral X-ray films of the knee joint of the affected limb on the second day after the operation; **e**-**f** One year after the operation, the patient’s anterior and lateral X-ray films of the knee joint; **g**-**h** functional appearance of the knee joint 1 year after the operation

**Fig. 5:**
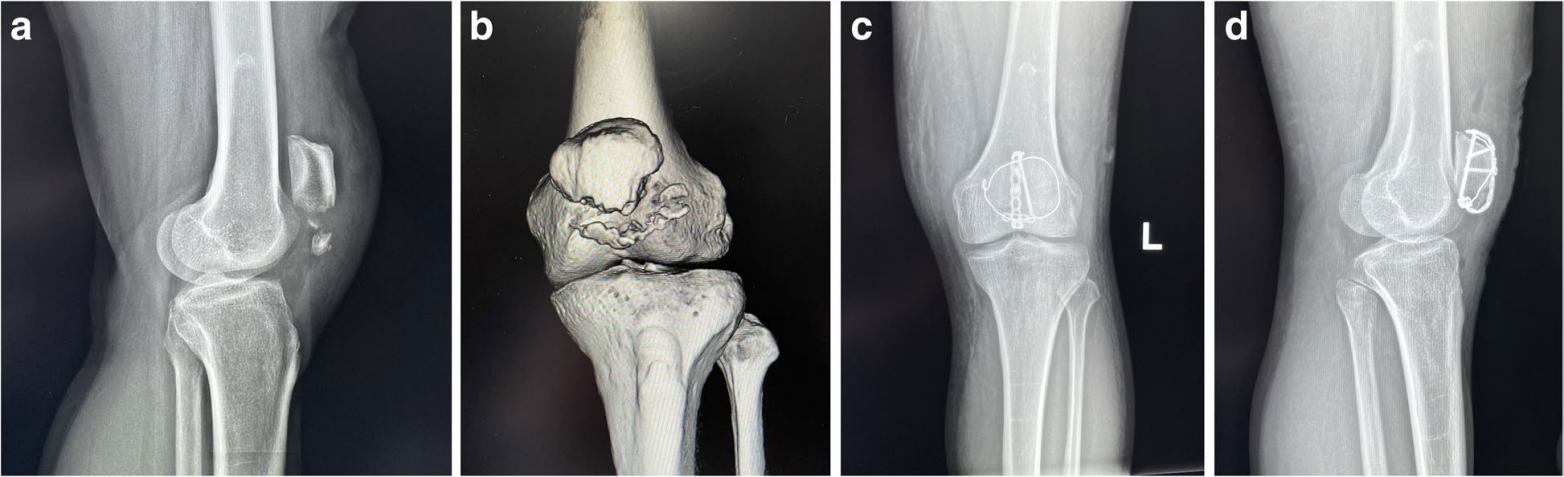
A 46-year-old female patient with distal patellar fracture. **a**-**b** Preoperative three-dimensional CT and lateral X-ray films of the knee joint; **c**-**d** anteroposterior and lateral X-ray films of the knee joint of the affected limb on the second day after the operation

Two typical cases are shown in Figs. 4 and 5.

## Discussion

Although inferior pole fractures of the patella do not affect the articular cartilage surface, they destroy the knee extension mechanism and require open reduction and internal fixation. The purpose of surgical treatment of patellar fracture is to restore the function of the quadriceps femoris, reconstruct the knee extension mechanism and promote early functional exercise. Different from other types of patellar fractures, inferior pole fracture fragments are usually comminuted and small, so they are difficult to effectively reduce and fix. Although TBW technology can convert the tension of patellar fracture displacement into compressive force so that the fracture can bear high tension load and promote fracture healing [14, 15], some studies have shown that the curative effect of TBW in patellar comminuted fractures is poor [16]. The more comminuted the fracture, the worse the union, which means that there may be some risks in its application in inferior pole fractures. Removal of patella fragments and repair of the patella tendon are considered to be the last choice for comminuted fractures of the inferior pole of the patella [17]. To address the challenge of reduction and fixation of comminuted fractures of the inferior pole of the patella, many new fixation techniques have been applied, including plate fixation [18, 19].

Previous biomechanical studies have shown that plate fixation achieves better fixation strength than TBW in patellar fractures [20–22]. Therefore, for elderly patients with comminuted patellar fractures, revision surgery, and severe osteoporosis, plate fixation can be considered, and inferior pole fracture is no exception. Plate fixation can not only increase the fixation strength of the TBW but also has minimal risk of internal fixation breakage and failure [23]. The basket plate has achieved encouraging clinical outcomes for fractures of the inferior pole of the patella [9, 10], but it has not been widely used, which may be related to its complex design or the cumbersome associated surgical procedures. Jang et al. [23] have also achieved good clinical results by using hook plates to fix various types of patellar fractures, but they are still fixed directly on the comminuted fracture fragment, which may have a certain risk. Zhu et al. [24] The operation method was chosen according to the degree of comminution of the inferior pole fracture of the patella. For fractures with a maximum transverse diameter of the lower pole of the patella less than 5 mm or fracture that are particularly comminuted, the fragments are sutured together with an absorbable intertwined suture, and then a micro steel plate is placed at the inferior pole of the patella and fixed with TBW. For large fracture fragments of the inferior pole of the patella, microplates and screws are directly fixed at the lower pole of the patella. However, for comminuted fractures of the inferior pole, this technology may involve a complex operation.

Our fixation concept comes from the concept of “drop anchor” (sailing term), which can generate traction on the ship and anchor the hull. The “anchor loop fixation device” in this study is based on clinical needs. The T-shaped plate is used as the anchor body, which is inserted in reverse from below the patellar tendon, and the patellar tendon and the fracture block at the lower pole of the patella are directly dragged and pulled to reduce and fix the fracture. At the same time, the T-shaped plate shaped by the long axis of the patella is used as the anchor body, and the holes on both sides of the disal transverse arm are used as loops through which to thread steel wires or cables. A three-dimensional multidirectional traction fixing device is formed. To ensure that the avulsion fracture of the lower pole of the patella is firmly pressed on the fracture end by the T-end of the steel plate, the continuity of the patellar tendon is reconstructed to restore the knee extension mechanism. Our fixation technique has the following advantages. First, the fracture fragment of the inferior pole of the patella is usually comminuted. Most of the previous plate fixation techniques use screw fixation on the comminuted fracture fragment, which may further increase the difficulty of reduction and fixation. Our fixation method avoids this perfectly. We only need to press the plate on the fracture fragment after reduction of the inferior pole fracture. Meanwhile, in our clinical practice, we found that the fixation strength of the intraoperative movable knee joint was reliable. Second, our technology can address the overall dragging and traction of the patellar tendon and the lower pole fracture block, directly bridge and restore the continuity of the knee extension mechanism, effectively avoid operation failure caused by insufficient fixation strength for the lower pole fracture block, and allow the patient to perform knee flexion and extension soon after the operation. Third, in the process of fixing the plate, we placed the unicortical locking screws from front to back. Similarly, patella fractures usually open forward due to the traction of the patellar tendon. We placed the plate on the front side, that is, the tension side, to further increase the fixation strength. Finally, we chose a T-shaped plate for shaping, which can not only better wrap the inferior pole fracture block but also be combined with the cerclage technique. Through cerclage, while further pressurizing the broken end of the fracture, the left- and right-side fixation of the T-shaped plate is made more reliable, preventing the displacement and detachment of fracture fragments.

Our results showed that none of the patients experienced implant failure or any infection. At least 90° of knee flexion was obtained 6 weeks after the operation, and the fractures of all patients achieved union within 12 weeks. At the last follow-up, all patients achieved satisfactory knee function, and no patients reported internal fixation-related irritation symptoms. The original intention of our development of this technology was to address fixation of small fractures of the lower pole of the patella. Because this technology can offset the traction effect of part of the patellar tendon on the fracture of the lower pole, the fixation is more reliable, and internal fixation cutting is relatively low. In this study, the diameter of the lower pole fracture block in 21 patients was 9.0 ~ 13.2 mm, with an average of 10.82 1.39 mm, and good clinical results were obtained. However, when using this technique to fix the fracture of the inferior pole of the patella, it should be noted that the plate fixation itself cannot compress the broken end of the fracture, so we need to compress the main fracture pieces through clamps and cerclage fixation to play a role in compression. Our plate is placed in the front of the patella, and its opposite side is the patellofemoral articular surface. Therefore, we can only use monocortical screw fixation to avoid damaging the patellofemoral articular surface.

Our study demonstrated a new technique for the fixation of inferior patellar fractures that is mainly used for the fixation of patellar marginal fractures for which TBW may not be adequate. The limitations of our study are its retrospective nature, small sample size, and lack of a control group. Although the operations of all patients were performed by the same professional orthopaedic doctor, because this is a novel technology, with the learning and progress of surgical technology, the surgical technology available for the more recently treated patients may have been better. In addition, we lack biomechanical research on this new fixation technology, and we look forward to addressing this deficiency in follow-up research.

## Conclusion

We used a modified T-shaped plate combined with cerclage as an effective fixation technique for inferior patellar fracture. It has the advantages of being simple to use, offering reliable fixation strength and allowing early functional exercise. It yields good knee function for patients with inferior patellar fractures after surgery.

## Data Availability

The data that supported the findings of this study are available on request from the corresponding author. The data are not publicly available due to privacy or ethical restrictions.
